# NHR-49 Transcription Factor Regulates Immunometabolic Response and Survival of Caenorhabditis elegans during Enterococcus faecalis Infection

**DOI:** 10.1128/IAI.00130-20

**Published:** 2020-07-21

**Authors:** Madhumanti Dasgupta, Meghana Shashikanth, Anjali Gupta, Anjali Sandhu, Atreyee De, Salil Javed, Varsha Singh

**Affiliations:** a Department of Molecular Reproduction, Development and Genetics, Indian Institute of Science, Bangalore, Karnataka, India; b Centre for Biosystems Science and Engineering, Indian Institute of Science, Bangalore, Karnataka, India; Georgia Institute of Technology School of Biological Sciences

**Keywords:** *Caenorhabditis elegans*, *Cryptococcus neoformans*, *Enterococcus faecalis*, *Pseudomonas aeruginosa*, fatty acids, immune response, metabolism, nuclear hormone receptor, nutritional immunity

## Abstract

Immune response to pathogens is energetically expensive to the host; however, the cellular source of energy to fuel immune response remains unknown. In this study, we show that Caenorhabditis elegans exposed to pathogenic Gram-positive and Gram-negative bacteria or yeast rapidly utilizes lipid droplets, the major energy reserve. The nematode’s response to the pathogenic bacterium Enterococcus faecalis entails metabolic rewiring for the upregulation of several genes involved in lipid utilization and downregulation of lipid synthesis genes. Genes encoding acyl-CoA synthetase ACS-2, involved in lipid metabolism, and flavin monooxygenase FMO-2, involved in detoxification, are two highly upregulated genes during E. faecalis infection. We find that both ACS-2 and FMO-2 are necessary for survival and rely on NHR-49, a peroxisome proliferator-activated receptor alpha (PPARα) ortholog, for upregulation during E. faecalis infection. Thus, NHR-49 regulates an immunometabolic axis of survival in C. elegans by modulating breakdown of lipids as well as immune effector production upon E. faecalis exposure.

## Abstract (precis)

Immune response to pathogens is energetically expensive to the host; however, the cellular source of energy to fuel immune response remains unknown. In this study, we show that Caenorhabditis elegans exposed to pathogenic Gram-positive and Gram-negative bacteria or yeast rapidly utilizes lipid droplets, the major energy reserve. The nematode’s response to the pathogenic bacterium Enterococcus faecalis entails metabolic rewiring for the upregulation of several genes involved in lipid utilization and downregulation of lipid synthesis genes.

## INTRODUCTION

Host response to microbial infection requires a rapid rewiring of transcriptional programs to enable the production of antimicrobials to provide resistance and to activate repair machinery and anti-inflammatory pathways to provide tolerance. Both resistance and tolerance mechanisms require reallocation of resources (1, 2). Constitutive and inducible innate defenses are present in all multicellular animals. The latter has a high energy cost, and it entails a systemic inflammatory response composed of increased production of acute-phase proteins or equivalent effectors, changes in energy and nutrient metabolism, anorexia, and fever in mammals (3). Fast-living species with high reproduction rates and short life spans rely heavily on innate inducible defenses and incur high cost during infection, forcing a trade-off with other life history traits such as reproduction (4). In Caenorhabditis elegans, brood size is inversely correlated with the immune response to pathogenic microbes (5), suggesting that inducible defenses may pose a high cost in this short-lived nematode as well.

C. elegans, in its natural habitat, is exposed to a variety of microbes, many of which are pathogenic. Immune response to pathogenic microbes entails accelerated production of a large number of immune effectors such as lectins, antibacterial factors, lysozymes, saposins, caenacins, and neuropeptide-like proteins (6–8). The nematode’s defense response is mounted as early as 4 h and continues for at least 24 h postexposure (8). Even within this short period of 4 h, there is a dramatic increase in immune effector expression. As many as 30 immune effectors with antimicrobial or xenobiotic detoxification function are induced from 4- to 100-fold when C. elegans is infected by Staphylococcus aureus (7). Rapid induction of immune effectors likely poses an increased demand for energy reserves in nematodes. However, the source of energy to fuel immune response remains to be investigated.

Lipid droplets (LDs) are membrane-enclosed organelles for storing energy in the form of neutral lipids such as triacylglycerol (TAG) and cholesterol esters. During fasting, neutral LDs are broken down by lipases to release free fatty acids, which then undergo beta-oxidation to generate acetyl coenzyme A (acetyl-CoA). This, in turn, is utilized in the tricarboxylic acid (TCA) cycle or in glyoxylate shunt (present in plants, nematodes, bacteria, and fungi) to generate energy and amino acid precursors, or acetyl-CoA can be utilized for gluconeogenesis. The utilization of neutral lipids is a highly orchestrated process, controlled by a complex network of conserved signaling pathways which involves many metabolic sensors and transcription factors (9–12). In C. elegans, LDs are utilized rapidly during nutritional, oxidative, and cold stresses (13–16). Polyunsaturated fatty acids (gamma-linoleic acid and stearidonic acid) are required for C. elegans’ immune response to Pseudomonas aeruginosa (17), while the stearoyl-CoA dehydrogenase enzyme, required for monounsaturated fatty acid synthesis, also regulates immunity (18). Although these studies suggest an important role of unsaturated fatty acids in the immune response, it is not yet clear how pathogenesis regulates fat metabolism in the host and if LDs are altered/utilized during infection.

In this study, we asked if C. elegans metabolism influences the immune response of the host to pathogenic microbes. We find that all five pathogens tested—Enterococcus faecalis, S. aureus, P. aeruginosa, Salmonella enterica serovar Typhimurium, and Cryptococcus neoformans—induce utilization of LD stores in the C. elegans intestine but with different time kinetics. To understand the regulation of immunometabolism in the host during infection, we utilized E. faecalis. We demonstrate that E. faecalis induces the transcription of genes encoding enzymes involved in neutral lipid breakdown and also suppresses key enzymes involved in neutral lipid synthesis in C. elegans. We show that E. faecalis-induced lipid breakdown, as well as immune effector production, is dependent on a nuclear hormone receptor, NHR-49. An increase in neutral lipids boosts the survival of C. elegans on E. faecalis in an NHR-49-dependent manner.

## RESULTS

### Exposure of C. elegans to pathogens induces transcriptional upregulation of lipid breakdown pathways and immune effectors.

**FIG 1:**
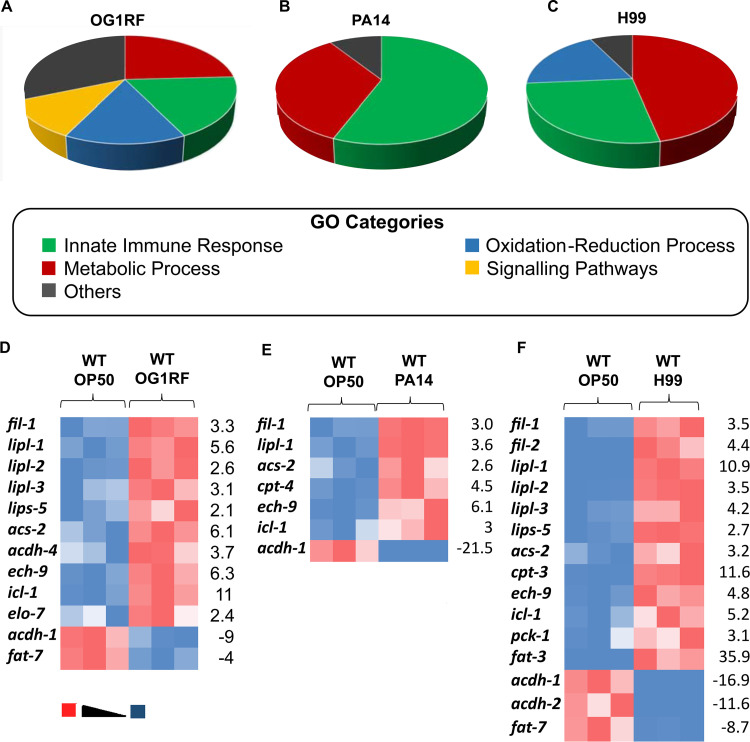
Exposure of C. elegans to pathogenic microbes induces distinct gene expression programs. Pie charts for GO categories enriched in C. elegans fed for 8 h on E. faecalis OG1RF (A), P. aeruginosa PA14 (B), and C. neoformans H99 (C). The number of genes in each GO category is indicated. Heat maps of expression of lipid metabolism genes in C. elegans fed OG1RF (D), PA14 (E), and H99 (F) compared to that of animals fed OP50. Fold change in expression of some of the dysregulated genes is indicated at the right side of each heat map.

To find evidence of energy utilization by the host during infection, we characterized the transcriptional response of C. elegans to different pathogens, E. faecalis OG1RF, P. aeruginosa PA14, and C. neoformans H99, by RNA sequencing. RNA sequencing of animals fed Escherichia coli OP50 was performed as a control. Exposure of adult nematodes to OG1RF, PA14, and H99 led to a >2-fold (*P* value < 0.05) upregulation of 1,460, 558, and 744 genes, respectively (see Table S1 in the supplemental material). There were 104 genes (see Fig. S1) induced by all three pathogens (highlighted in Table S1). These contained Gene Ontology (GO) categories such as innate immune or defense response, metabolic processes, oxidation-reduction processes, and cell signaling pathways (represented in Fig. 1A to C). During OG1RF infection, 57 immune effectors (see Fig. S2 for immune effectors) and 75 metabolic genes were upregulated, while 35 immune effectors and 60 metabolic genes were upregulated during H99 infection, suggesting that transcripts for metabolic genes represented an appreciable proportion of C. elegans response at 8 h of infection. In the case of PA14 infection, the number of induced putative immune effectors (59) was higher than the number of metabolic genes (36). Genes in several GO categories, such as regulation of cell shape, anion transport, fucosylation, signal transduction, and protein phosphorylation (in OG1RF-infected animals), chromatin silencing and MAPK pathway (in PA14-infected animals), and somatic sex determination, germ line sex determination, chloride transport, and sphingolipid metabolism (in H99-infected animals), were also altered and were categorized as “others” in the pie charts (Fig. 1A to C). Of the transcripts in the metabolism category, many genes involved in sequential steps of lipid breakdown were dysregulated strongly during E. faecalis infection, mildly during C. neoformans infection, and less so during P. aeruginosa infection (Fig. 1D to F). Taken together, our analyses of C. elegans response at 8 h of infection with bacterial and fungal pathogens suggested activation of the transcriptional programs for immune effector production as well as lipid breakdown.

### Infection initiates a transcriptional program for neutral lipid utilization in C. elegans.

**FIG 2:**
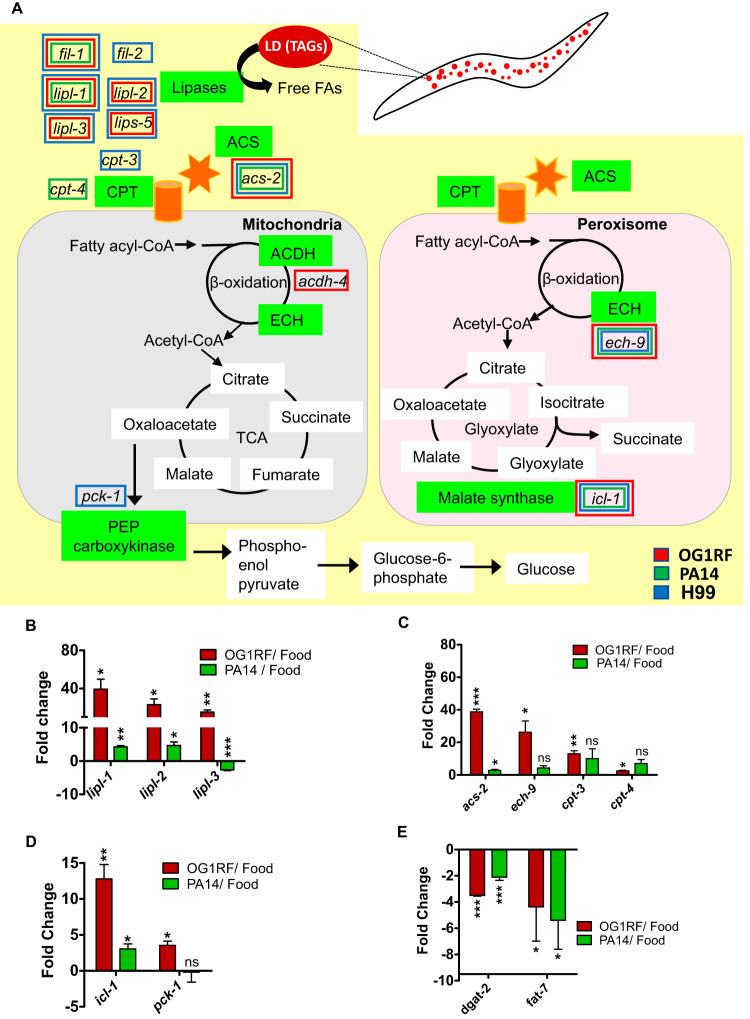
Lipid breakdown pathways are activated in C. elegans upon infection. (A) Schematic of lipid droplet hydrolysis in C. elegans. Each green box represents a class of enzymes upregulated during infection. Transcripts that are induced upon infection have been highlighted with colored boxes. Color indicates specific pathogen (red, OG1RF; green, PA14; blue, H99). qRT-PCR analysis of genes involved in lipid breakdown (B), beta-oxidation (C), glyoxylate shunt and gluconeogenesis (D), and lipid synthesis (E) in C. elegans L4 fed OG1RF or PA14 for 8 h with respect to expression in animals fed OP50. Significance was calculated by unpaired *t* test: *, *P* ≤ 0.05; **, *P* ≤ 0.01; ***, *P* ≤ 0.001; ns, not significant.

We looked for evidence of metabolic dysregulation during C. elegans infection with a special focus on lipid breakdown enzymes. Dysregulation observed in RNA sequencing (RNA-Seq) data is highlighted in the schematic in Fig. 2A (see Table S1 and Fig. 2 for fold changes). We confirmed the upregulation of transcripts for lipid breakdown enzymes in animals infected with OG1RF and PA14 by using quantitative real-time PCR (qRT-PCR) (Fig. 2B to E). Lipid breakdown requires a sequential breakdown of triacylglycerol (TAG) to fatty acids and breakdown of individual fatty acids to acetyl-CoA. Transcripts for lipases involved in TAG breakdown, *lipl-1*, *lipl-2*, and *lipl-3*, were induced in OG1RF- and PA14-infected animals (Fig. 2B), except for *lipl-3*, which was significantly downregulated in animals fed PA14. Enzymes involved in beta-oxidation such as *acs-2*, *ech-9*, *cpt-3*, and *cpt-4* were induced in OG1RF-infected animals. Among these 4 transcripts, only *acs-2* was induced in PA14-infected animals (Fig. 2C). Surprisingly, the induction of all the transcripts was muted in animals infected with PA14 as opposed to animals infected with OG1RF (Fig. 2B to E), indicating a pathogen-specific pattern of metabolic dysregulation. Overall, the transcriptome analyses showed that infection by OG1RF, PA14, or H99 induces a broad transcriptional response to facilitate sequential steps of lipid hydrolysis in C. elegans.

We wanted to examine the fate of acetyl-CoA generated by fatty acid beta-oxidation. We found a significant increase in the transcript level of isocitrate lyase 1 (ICL-1)/malate synthase, the rate-limiting enzyme of the glyoxylate shunt, by OG1RF as well as PA14 infection (Fig. 2D), indicating the activation of this shunt which can facilitate the production of ATP, reducing equivalents and amino acid precursors during infection. Here also, the level of *icl-1* induction was much lower during PA14 infection as opposed to that during OG1RF infection. We also found that the transcript for rate-limiting enzyme of gluconeogenesis, PEP carboxykinase encoded by *pck-1*, was also upregulated ∼3-fold in OG1RF-fed animals but not in those fed PA14 (Fig. 2D), suggesting infection-specific metabolic reprogramming early during infection.

While lipid breakdown was initiated during infection, we found evidence for the reduction in lipid synthesis in OG1RF-infected as well as in PA14-infected animals. We observed 3- to 4-fold downregulation of transcripts for key fatty acid desaturase *fat-7* and diacylglycerol-acyltransferase *dgat-2*, encoding the rate-limiting enzyme for lipid droplet synthesis in OG1RF-infected animals (Fig. 2E). *dgat-2* was downregulated 2-fold and *fat-7* was downregulated 4-fold in PA14-infected animals (Fig. 2E). Coordinated downregulation of lipid synthesis during the upregulation of lipid breakdown has been shown during starvation (12). In all, we found both quantitative and qualitative differences in metabolic responses in an infection-specific manner. Thus, the transcriptome analysis suggested that C. elegans exposed to the pathogen for 8 h engages in a metabolic response.

### Pathogenic bacteria and yeast induce neutral lipid depletion in C. elegans intestine during infection.

**FIG 3:**
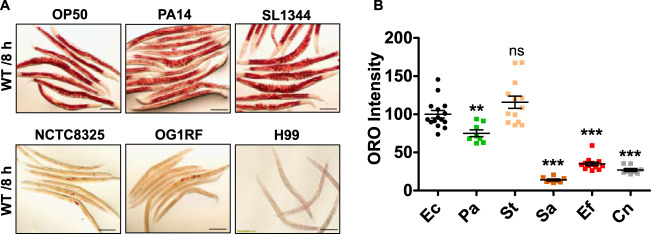
C. elegans exposed to pathogens undergoes lipid droplet depletion. (A and B) Images of ORO stained C. elegans and quantification in adult animals fed E. coli (OP50), P. aeruginosa (PA14), *S*. Typhimurium (SL1344), S. aureus (NCTC8325), E. faecalis (OG1RF), and C. neoformans (H99) for 8 h. Bars, 100 μm. Data are presented as means ± SEMs. *n* = 10 to 15 animals/condition/experiment.

Analyses of the nematodes' transcriptomes suggested that infection activates pathways involved in LD breakdown in C. elegans. To investigate the effect of transcriptional dysregulation on lipid stores, we tested the status of stored lipids in C. elegans during infection. We exposed C. elegans adults to pathogenic Gram-negative bacilli, P. aeruginosa PA14 and *S*. Typhimurium SL1344, to pathogenic Gram-positive cocci, S. aureus NCTC8325 and E. faecalis OG1RF, and to pathogenic yeast, C. neoformans H99, for 8 h. C. elegans that fed on P. aeruginosa showed a small decline in lipid droplet staining with oil red O (ORO), while C. elegans that fed on *S*. Typhimurium showed no change in LD stores (Fig. 3A and B). However, animals fed on C. neoformans, on S. aureus, or on E. faecalis showed nearly complete depletion of ORO-stainable lipids within 8 h of feeding (Fig. 3A and B). LD depletion began very early during E. faecalis infection and was observed within 2 h of feeding (see Fig. S3A and B). These experiments indicated that E. faecalis OG1RF (also S. aureus and C. neoformans) exposure induces rapid LD depletion in C. elegans intestine.

The depletion of neutral lipids upon exposure to OG1RF was confirmed using neutral lipid stain boron-dipyrromethene (BODIPY^493/503^) (Fig. S3C and D). Lynn et al. have shown that C. elegans mobilizes lipids to their germ line during nutrient and oxidative stress (14). To determine if germ line played any role in infection-induced LD depletion, we first studied the effect of the OG1RF diet on lipid stores in very young L4 larvae, before gametocyte development and proliferation. We found that lipid stores were depleted in young L4 larvae on the OG1RF diet (Fig. S3E and F). In the second approach, we prevented germ line proliferation by *cdc25.1* RNA interference (RNAi) and found that the OG1RF diet caused the depletion of lipid stores in these animals also (Fig. S3G and H). We found that heat-killed OG1RF also induced LD depletion in C. elegans, as did OG1RF exposure at 20°C (Fig. S3I to L). In all the preceding experiments, OG1RF was grown on nutrient-rich brain heart infusion (BHI) agar. To test for the effect of the bacterial growth medium, we exposed C. elegans to OG1RF grown on nematode growth medium (NGM) agar and observed LD depletion under this condition as well (Fig. S3M and N). Thus, OG1RF exposure induced LD depletion in C. elegans in a temperature-independent as well as germ line-independent manner.

We also examined carbohydrate content and protein content in OG1RF- and OP50-fed adult C. elegans (Fig. S4A and B). Although protein levels remained similar in OP50- and OG1RF-fed animals, we found that OG1RF-fed animals had ∼50% lower carbohydrate content than OP50-fed animals (Fig. S4A). Low carbohydrate content suggested that the energy contained in the carbohydrates was also utilized during E. faecalis infection of C. elegans. Loss of lipid and carbohydrates could result from poor feeding on an E. faecalis diet. We found a very small decline in pharyngeal pumping on OG1RF compared to that on OP50 (see Fig. S5A), suggesting that the animals were feeding on E. faecalis. Consistent with this observation, we found that the C. elegans intestinal lumen was full of E. faecalis, as we recovered an average of 20,000 cocci per animal in 8 h of feeding (Fig. S5B and C), suggesting that nematodes were able to ingest OG1RF. Taken together, our results suggested that C. elegans ingestion of OG1RF results in carbohydrate and lipid depletion.

### Acyl-CoA synthetase and NHR-49 transcription factor regulate survival of C. elegans during E. faecalis infection.

**FIG 4:**
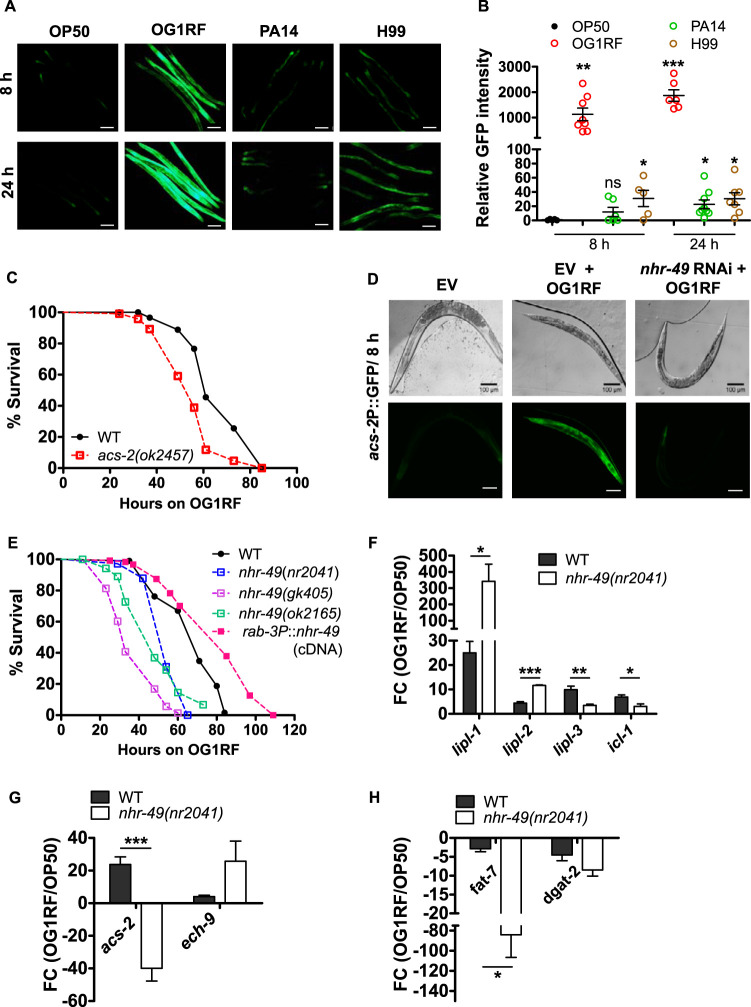
Metabolic response of C. elegans is linked with its survival upon E. faecalis infection. (A and B) Images and quantification of *acs-2*P::GFP animals fed OP50, OG1RF, PA14, and H99 for 8 and 24 h. Bars, 100 μm. (C) Kaplan-Meier survival curves of *acs-2(ok2457)* and WT animals exposed to OG1RF (*P* < 0.0001). (D) Effect of *nhr-49* RNAi on *acs-2*P::GFP fluorescence in animals upon OG1RF exposure for 8 h. Bars, 100 μm. (E) Kaplan-Meier survival curves of *nhr-49(nr2041)* (*P* < 0.0001 against WT), *nhr-49(gk405)* (*P* < 0.0001 against WT), *nhr-49(ok2165)* (*P* < 0.0001 against WT), *rab-3*P::*nhr-49* overexpression [*P* < 0.0001 against *nhr-49(nr2041)*], and WT animals exposed to OG1RF. qRT-PCR analysis of OG1RF-regulated expression of lipases and *icl-1* (F), *acs-2* and *ech-9* (G), and *fat-7* and *dgat-2* (H) in *nhr-49*(*nr2041*) animals compared to WT animals. Data are presented as means ± SEMs. Significance was calculated by unpaired *t* test: *, *P* ≤ 0.05; **, *P* ≤ 0.01; ***, *P* ≤ 0.001.

Of the 22 acyl-CoA synthetases encoded by the C. elegans genome, *acs-2* was induced >40-fold in E. faecalis-infected animals. For confirmation, we allowed infection of *acs-2*P::GFP transgenic animals with OG1RF, PA14, or H99 for 8 h and observed a robust induction of green fluorescent protein (GFP) expression (Fig. 4A and B) in a pathogen-specific manner, consistent with our RNA-Seq data. We found a 1,000-fold increase in the expression of GFP in animals infected by E. faecalis for 8 and 24 h. However, we found lower induction of the reporter in PA14- and H99-infected animals at both 8 h and 24 h, suggesting that acyl-CoA synthetases other than ACS-2 may help in lipolysis. Interestingly, we found that *acs-2*P::GFP was induced even by heat-killed E. faecalis, suggesting that a heat-stable factor of E. faecalis may induce lipid breakdown (see Fig. S6). Next, we checked whether ACS-2 is necessary for the survival of C. elegans upon E. faecalis infection. To test this, we examined the survival of wild-type and *acs-2(ok2457)* animals during E. faecalis infection. We found that *acs-2* mutants had enhanced susceptibility to E. faecalis infection compared to that of wild-type (WT) animals (Fig. 4C). This experiment suggested that beta-oxidation of fatty acid is necessary for survival during E. faecalis infection and mediates the immunometabolic response to infection.

Regulation of beta-oxidation enzyme ACS-2 is dependent on the function of a nuclear hormone receptor, NHR-49, an ortholog of human peroxisome proliferator-activated receptor α (PPARα) which regulates expression of other lipid metabolism enzymes as well (11, 19). To test whether NHR-49 regulates the expression of ACS-2 during infection, we performed RNAi of *nhr-49* in *acs-2*P::GFP animals before exposure to OG1RF. We observed that knockdown of *nhr-49* in the *acs-2*P::GFP reporter strain resulted in almost complete suppression of *acs-2*P::GFP induction by both live and heat-killed E. faecalis infection (Fig. 4D; Fig. S6). As a control, we tested the effect of SBP-1, a transcriptional regulator of lipid metabolism, but found that it did not affect *acs-2* reporter expression during E. faecalis infection (see Fig. S7). This experiment suggested that NHR-49 is essential for *acs-2* induction in C. elegans intestine during infection.

Next, we tested the role of NHR-49 in survival response during E. faecalis infection. We utilized three deletion alleles, *nhr-49(nr2041)*, *nhr-49(gk405)*, and *nhr-49(ok2165)* in survival assays and found that all the mutants showed enhanced susceptibility to E. faecalis infection compared to that of WT animals (Fig. 4E), consistent with the *nhr-49* RNAi phenotype shown earlier (20). Since intestine and hypodermis are major neutral lipid storage sites, we first tested the effect of NHR-49 in the intestine. Rescue of NHR-49 expression in the intestines of *nhr-49(nr2041)* animals did not completely rescue the enhanced susceptibility phenotype of *nhr-49* mutants (data not shown). This suggested that NHR-49 activity in other tissues might regulate the resistance of C. elegans to E. faecalis infection. We found that overexpression of NHR-49 under neuronal promoter *rab-3*P resulted in enhanced resistance of animals to E. faecalis infection (Fig. 4E), suggesting that neuronal function of NHR-49 is necessary for boosting survival upon E. faecalis infection.

To investigate alternate pathways of regulating LD mobilization upon infection, we examined the roles of 18 additional NHRs in regulating the survival of C. elegans fed OG1RF. These NHRs were previously reported to be induced during starvation and downregulated upon refeeding, suggesting their role in metabolism (21). However, none of these NHRs had any effect on the survival of animals during OG1RF infection (see Fig. S8). We hypothesized that either NHR-49 is essential for survival during all infection or NHR-49 has a context (infection)-specific role. Therefore, we tested the role of NHR-49 on survival of C. elegans during infection with five pathogenic microbes which induced lipid depletion. We observed that *nhr-49* RNAi inhibition had no appreciable effect on susceptibility to S. aureus and a very modest increase in susceptibility to PA14, SL1344, and H99 (see Fig. S9A to D). This indicated that *nhr-49* mutants were not sick but NHR-49 had a context-specific function in survival during infection of C. elegans with E. faecalis.

We also quantified *acs-2* expression in WT and *nhr-49(nr2041)* animals by qRT-PCR and found that the upregulation of *acs-2* transcript by OG1RF was entirely dependent on NHR-49 (Fig. 4G). In addition, we found that NHR-49 activity was also required for the upregulation of TAG lipase *lipl-3* and for the upregulation of glyoxylate shunt enzyme *icl-1* on the OG1RF diet (Fig. 4F). Surprisingly, we found that *nhr-49*(*nr2041*) mutant animals had elevated levels of transcripts for lipases, *lipl-1*, *lipl-2*, and *ech-9* (Fig. 4F and G), pointing to possible activation of a compensatory mechanism for lipid breakdown upon OG1RF exposure. We also analyzed the effect of NHR-49 on two lipid synthesis genes, *fat-7* and *dgat-2*, downregulated by infection. We found that OG1RF-mediated *fat-7* downregulation was dependent on NHR-49 (Fig. 4H).

### NHR-49-dependent immune effector FMO-2 is required for survival of C. elegans during infection.

**FIG 5:**
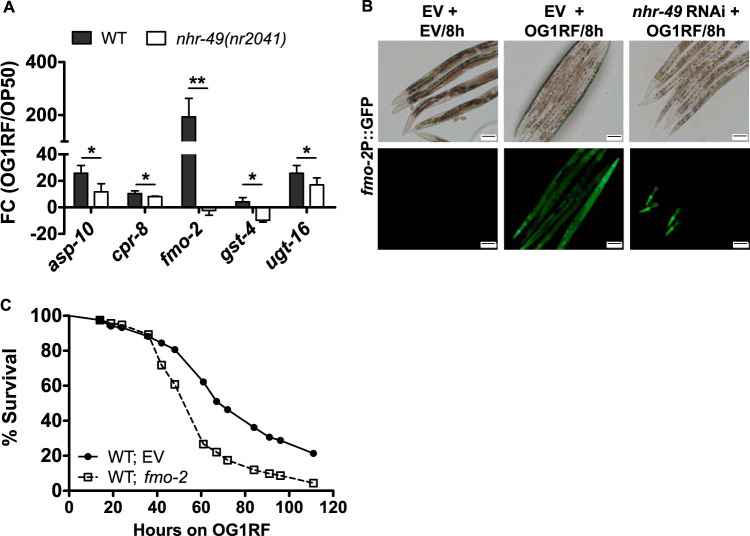
NHR-49-regulated immune effector, FMO-2, mediates survival of C. elegans upon E. faecalis infection. (A) qRT-PCR analysis of OG1RF-induced expression of immune effectors in *nhr-49(nr2041)* animals compared to that in WT animals. (B) OG1RF-induced expression of *fmo-2*P::GFP in WT and *nhr-49* RNAi animals. Bars, 100 μm. (C) Kaplan-Meier survival curves for WT and *fmo-2* RNAi animals exposed to OG1RF (*P* < 0.0001). In panel A, the data are presented as means ± SEMs. Significance was calculated by unpaired *t* test: *, *P* ≤ 0.05; **, *P* ≤ 0.01.

The requirement of NHR-49 during E. faecalis exposure suggested that this transcription factor might promote immune effector production. Several immune effectors, antimicrobial factors, and detoxification enzymes are upregulated during OG1RF infection in transcriptome analysis (see Table S1A). We validated the upregulation of ten of these transcripts in WT animals infected with E. faecalis for 8 h. Five transcripts, namely, UDP-glucuronosyltransferase (*ugt-16*), flavin monooxygenase (*fmo-2*), cysteine protease (*cpr-8*), aspartyl protease (*asp-10*), and glutathione *S*-transferase (*gst-4*), were not upregulated optimally in *nhr-49*(*nr2041*) animals exposed to OG1RF for 8 h (Fig. 5A; also see Fig. S10). Of these effectors, *fmo-2* showed a high induction (150-fold) upon OG1RF exposure. Using *fmo-2*P::GFP transgenic animals, we found that the reporter was induced dramatically in WT animals (Fig. 5B); however, *nhr-49* RNAi suppressed OG1RF-mediated induction of *fmo-2* expression in the intestine of C. elegans during E. faecalis infection (Fig. 5B). To test whether FMO-2 serves an immune effector function, we tested the survival of *fmo-2* RNAi animals upon infection with OG1RF. *fmo-2* RNAi caused enhanced susceptibility of C. elegans to E. faecalis infection, providing support for its role as an important immune effector necessary for survival (Fig. 5C). In all, our observations provided mechanistic insights into NHR-49-dependent mechanisms of survival.

### Neutral lipids boost C. elegans survival during E. faecalis infection.

**FIG 6:**
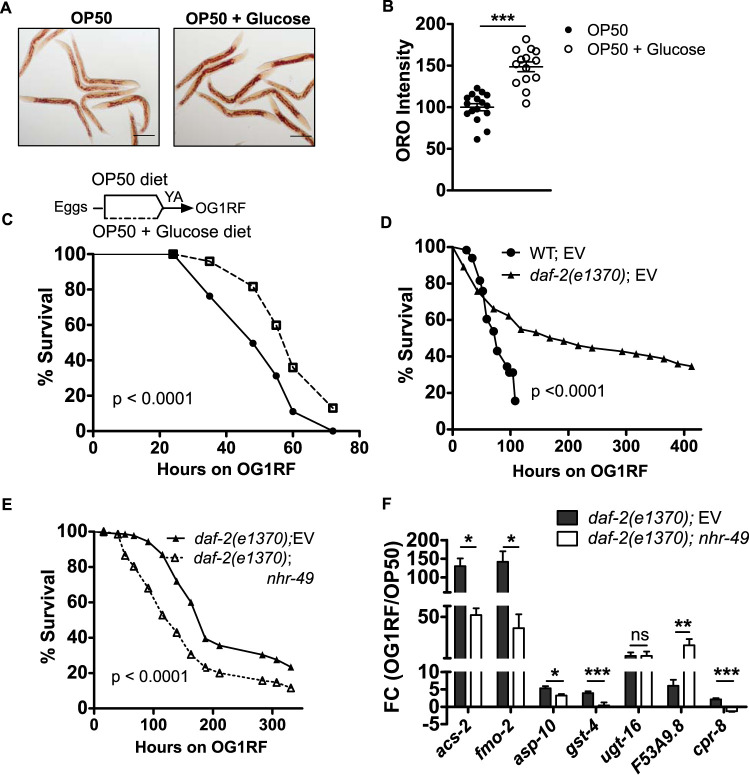
Lipids boost C. elegans survival during E. faecalis infection. (A and B) ORO staining and quantification of adult C. elegans fed an OP50 plus 10 mM glucose diet compared to those fed an OP50 diet. (C) Kaplan-Meier survival curves on OG1RF for animals from glucose-supplemented diet and normal diet (*P* < 0.0001). (D) Kaplan-Meier survival curves for WT and *daf-2(e1370)* animals exposed to OG1RF (*P* < 0.0001). (E) Kaplan-Meier survival curves for *daf-2(e1370)*;empty vector (EV) and *daf-2(e1370)*;*nhr-49* RNAi animals exposed to OG1RF (*P* < 0.0001). (F) qRT-PCR analysis of OG1RF-induced expression of *acs-2* transcript and immune effector transcripts in *daf-2(e1370)* animals with *nhr-49* RNAi relative to that with vector RNAi. In panels B and F, significance was calculated by unpaired *t* tests: *, *P* ≤ 0.05; **, *P* ≤ 0.01; ***, *P* ≤ 0.001.

Both the glyoxylate shunt and gluconeogenesis were activated in OG1RF-infected animals, allowing us to reason that energy stored in neutral lipids could boost the immune response during infection. We hypothesized that animals with increased LD content can mount a more effective immune response when exposed to OG1RF. To increase the neutral lipid content in C. elegans intestine, we utilized a regime of 10 mM glucose supplementation for only 2 days during C. elegans larval development, as shown previously (22). Glucose supplementation, prior to infection, led to 30% more ORO staining in adults than in the control (Fig. 6A and B). Glucose-supplemented adults, with more ORO-stained lipids, showed a modest but significant increase in survival upon chronic exposure to OG1RF than animals with normal LD stores (Fig. 6C). This indicated that an increase in lipid stores in C. elegans boosts host survival.

To confirm that the utilization of stored lipids can boost host survival, we also utilized a genetic model of high fat in C. elegans. Mutation in *daf-2*, the gene encoding insulin-like receptor, causes a high-body fat phenotype (23). *daf-2(e1370)* animals are also resistant to a number of abiotic stresses (24) and to microbial pathogens, including OG1RF and PA14, as shown by us and others (25, 26). We found that *daf-2(e1370)* animals were more resistant than WT animals to OG1RF (Fig. 6D), as expected. To check if immunometabolism contributed to enhanced resistance, we studied the effect of *nhr-49* RNAi in *daf-2(e1370)* animals. We found that *daf-2(e1370)* animals with *nhr-49* RNAi were more susceptible to death by OG1RF than animals with control RNAi (Fig. 6E), suggesting that NHR-49 contributes to enhanced resistance of *daf-2* mutants. Also, *nhr-49* RNAi dampened the induction of *acs-2* in *daf-2(e1370)* animals exposed to OG1RF (Fig. 6F). Next, we tested whether NHR-49 activity regulates the expression of immune effectors in *daf-2(e1370)* animals. Indeed, the expression of *fmo*-2, *asp-10*, *cpr-8*, and *gst-4* transcripts were lower in *daf-2(e1370)* animals with *nhr-49* RNAi than those with vector control (Fig. 6F). Some of these transcripts were also altered at the basal levels in *daf-2(e1370)* animals (see Fig. S11). Thus, our data indicated that NHR-49 regulated both the metabolic response and the immune effector-dependent protective response in *daf-2* mutants exposed to E. faecalis. Taken together, our data suggested that an increase in the neutral lipid stores of C. elegans enhances its survival on E. faecalis in an NHR-49-dependent manner, underscoring immunometabolism as a major contributor to enhanced survival.

### Metabolic response to E. faecalis diet is conserved across roundworms.

**FIG 7:**
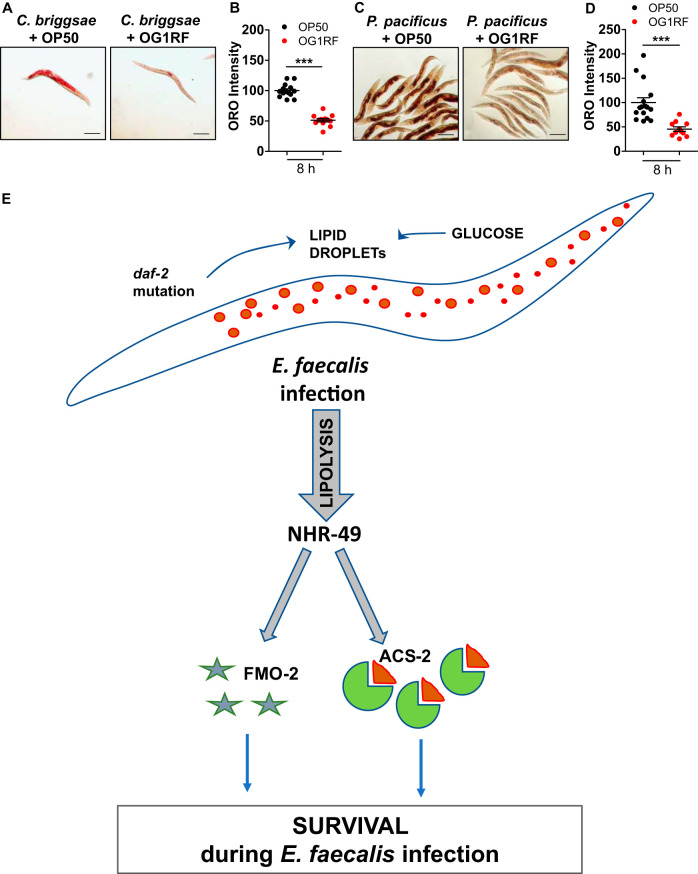
Immunometabolic response in C. elegans toward E. faecalis. (A and B) ORO staining and quantification of adult *Caenorhabditis briggsae* AF16 animals fed an OG1RF diet for 8 h. (C and D) ORO staining and quantification of adult *Pristionchus pacificus* PS312 animals fed an OG1RF diet for 8 h. The data are presented as means ± SEMs. *n* = 10 to 15 animals/condition/experiment. (E) Model of immunometabolic response of C. elegans to E. faecalis: animals fed on E. faecalis undergo lipolysis, modulated by nuclear hormone receptor, NHR-49. Expression of beta-oxidation enzyme acyl-CoA synthetase (ACS-2) and immune effector flavin monooxygenase (FMO-2), regulated by NHR-49, is crucial for survival during infection. Increase in lipid droplets via glucose supplementation and/or in *daf-2* mutation provides resistance to infection.

Gram-positive cocci are common in nematode habitats, and it is conceivable that LD mobilization is conserved in other nematodes. We tested the effect of OG1RF diet on two additional members of family Rhabditidae, Caenorhabditis briggsae and Pristionchus pacificus (27). OG1RF feeding caused the depletion of lipid stores in both these species within 8 h of feeding (Fig. 7A to D).

Taken together, our study shows that the utilization of neutral lipids is a conserved response of C. elegans and other roundworms to E. faecalis. Investigation of events during E. faecalis infection of C. elegans led us to propose a model for a host immunometabolic response to infection (Fig. 7E). This response is partly orchestrated by nuclear hormone receptor NHR-49, which facilitates the regulation of key enzymes involved in the sequential breakdown of neutral lipids (LIPL-3), and acetyl-CoA utilization (ACS-2) via glyoxylate shunt (ICL-1). Beta-oxidation enzyme ACS-2 also regulates the survival response in animals during E. faecalis infection. Our study reveals another role of NHR-49 during infection wherein it regulates the production of many immune effectors, of which, FMO-2 directly regulates survival. The NHR-49-regulated immunometabolic axis is also functional in a high-fat mutant and contributes to its enhanced resistance during E. faecalis infection.

## DISCUSSION

In this study, we show that exposure of C. elegans to pathogenic microbes induces a rapid rewiring of the metabolism in C. elegans which impinges on neutral lipid breakdown. An increase in neutral lipids boosts the survival of nematodes during E. faecalis infection. We find that acyl-CoA synthetase, important for facilitating beta oxidation of fatty acids, is necessary for survival on E. faecalis specifically, underscoring the importance of lipid breakdown. Both basal and inducible *acs-2* activities are dependent on the NHR-49 transcription factor, which is necessary for survival as well. NHR-49 is also required for the expression of protective immune effectors such as FMO-2 during E. faecalis infection. Thus, NHR-49 regulates the immunometabolic response which plays a key role in C. elegans-E. faecalis interactions (see graphical abstract, Fig. S12).

This study revealed that neutral lipid utilization is a conserved response of C. elegans to infection by different pathogens, with important context-specific differences. Induction levels of many transcripts were quite different for at least two classes of pathogens used in this study, Gram-positive bacterium E. faecalis and Gram-negative bacterium P. aeruginosa. E. faecalis infection resulted in higher fold induction of lipases and beta-oxidation genes than fold inductions observed in animals infected with P. aeruginosa. E. faecalis infection also resulted in a 13-fold induction of transcripts for the rate-limiting enzyme of the glyoxylate pathway, malate synthase, *icl-1*, and a 3-fold induction in PEP carboxykinase, *pck-1*, whereas P. aeruginosa infection resulted in a 3-fold induction of *icl-1* but no induction in *pck-1*. Our survey of previous C. elegans-microbe interaction studies allowed us to find evidence of lipid breakdown in C. elegans interaction with E. faecalis strain MMH594 (28), with S. aureus (7), and with P. aeruginosa (8). Our finding that lipid utilization is initiated within 2 h of feeding on E. faecalis is intriguing. Such a rapid metabolic response in C. elegans could result from differences in the digestibility of different microbes in the alimentary canal of C. elegans. The lysozyme-resistant nature of the cell walls of Gram-positive bacteria, including E. faecalis and S. aureus (29, 30), but lysozyme-susceptible nature of those of Gram-negative bacteria such as E. coli, *S*. Typhimurium, and P. aeruginosa (31) suggests better digestibility of Gram-negative bacteria by C. elegans who employ a battery of lysozymes for digestion. Poor digestibility of cocci in the C. elegans alimentary canal likely contributes to a rapid LD depletion response on the cocci diet compared to that with the equally pathogenic P. aeruginosa. Intrinsic differences in molecular features of pathogenesis by different pathogens could also contribute to altered kinetics of lipid depletion across infections. P. aeruginosa colonizes C. elegans intestine slowly and releases virulence-related membrane vesicles, whereas S. aureus colonizes the intestine rapidly, causing rapid loss of intestinal microvilli (7). This raises the possibility that the nematode’s cost of mounting an immune response might be higher in the case of infection with Gram-positive bacteria, prompting the utilization of neutral lipids early on.

We found certain similarities between our study and those studying starvation responses in C. elegans (11, 12, 32). These included upregulation of transcripts for enzymes involved in lipid breakdown. Of particular interest is isocitrate lyase, ICL-1, the rate-limiting enzyme of the glyoxylate pathway. Transcripts for this enzyme are induced during fasting, in dauers, and in *daf-2* mutant animals (32, 33). RNAi of *icl-1* (also called *gei-7*) leads to suppression of the enhanced life span of *daf-2* and *eat-2* mutants. The glyoxylate pathway produces less NADH than the TCA cycle, and this is believed to protect C. elegans from reactive oxygen species (ROS). It is important to note that ROS has also been linked to the death of the nematodes during E. faecalis infection (34). Additionally, NHR-49 is also activated during organic peroxide treatment and causes induction of cytoprotective factors, including FMO-2 (35, 36).

LD homeostasis entails a balance between lipid breakdown and synthesis. The homeostasis is maintained by conserved molecular players such as SREBP (SBP-1), PPARα (NHR-49), TOR, AMPK and hexosamine. Nuclear hormone receptor NHR-49, is a key regulator of the metabolic response induced in C. elegans during starvation (11, 12). It was, therefore, not altogether surprising that C. elegans was able to coopt NHR-49 to facilitate lipid breakdown and glyoxylate shunt during E. faecalis infection. More surprising was the fact that it also regulated immune effectors required to control E. faecalis infection. The effect of *nhr-49* knockdown on the susceptibility to E. faecalis infection was reported earlier, although the mechanism for survival was not elucidated (20). The study reported that NHR-49 did not positively regulate beta-oxidation enzyme *acs-2*. Our RNA-Seq analysis, qPCR data, and *acs-2*P::GFP reporter expression at 8 and 24 h of exposure clearly showed that NHR-49 positively regulates E. faecalis-induced upregulation of *acs-2* as well as of *lipl-3* and *icl-1*. We found that during E. faecalis infection, NHR-49 regulates two distinct sets of effectors: (a) lipid metabolism enzymes and (b) immune effectors. Of these immune effectors, aspartyl protease ASP-10, cysteine protease CPR-8, UDP-glucuronosyltransferase UGT-16, glutathione *S*-transferase GST-4, and flavin monooxygenase FMO-2, we find that FMO-2 is a direct regulator of C. elegans survival on E. faecalis. Recent reports indicate that NHR-49 can regulate expression of *gst-4* (35, 36) and *fmo-2* (35–37) in other contexts. Also, we show for the first time that ACS-2, an enzyme whose expression is transcriptionally regulated by NHR-49, is a direct regulator of survival response in C. elegans, specifically during E. faecalis infection. This study provides NHR-49 as the first example of a metabolic regulator engaged in immune effector production during E. faecalis infection.

We found that NHR-49 is required for enhanced survival of *daf-2* animals who are stress resistant and long lived. NHR-49 was necessary for enhanced expression of *acs-2*, *fmo-2*, and other effectors in *daf-2* mutants. To our surprise, NHR-49 was not essential for survival in all infections, ruling out the possibility that *nhr-49* animals were just sick or less fit under stresses of various kinds. NHR-49 appeared to have a minor role in survival on P. aeruginosa and C. neoformans and no role on S. aureus. However, NHR-49 was required for the survival of C. elegans on slow-killing bacterium *S*. Typhimurium. Interestingly, overexpression of *nhr-49* under a neuronal promoter, *rab-3*, resulted in enhanced resistance phenotype during E. faecalis infection. These data are consistent with the notion that NHR-49 works in a nonautonomous manner to regulate metabolism, including basal as well as fasting levels of *acs-2* (38). Our data suggest that NHR-49 may function in the neurons to regulate survival. It is tempting to think that NHR-49 might be regulated by microbe-specific cues or patterns of pathogenesis, but the identity of such patterns remains to be established. These unknown cues might activate NHR-49 and other pathways for neutral lipid breakdown. In an effort to find other regulators of immunometabolism, we studied additional NHRs implicated in starvation and refeeding responses (21). Surprisingly none of the 18 NHRs examined regulated nematode’s survival upon E. faecalis infection.

Recent studies have highlighted the importance of lipids in C. elegans during infection. Polyunsaturated fatty acids are required for survival of animals against P. aeruginosa infection through the regulation of immune response genes (17). Along the same lines, studies have shown that defects in the production of oleate, a monounsaturated fatty acid, result in the dampened immune response during infection by P. aeruginosa, E. faecalis, and Serratia marcescens (18). Lipid droplet dyshomeostasis was recently reported during P. aeruginosa infection in C. elegans. (39). Consistent with these findings, we show that the immunometabolic response of C. elegans to E. faecalis is a major regulator of survival.

Bacterial pathogens Chlamydia trachomatis and Mycobacterium tuberculosis are found to be intimately associated with host LDs (40, 41). In hepatitis C virus infection, host LDs serve as platforms for viral assembly (42, 43), while LDs bound to histones are proposed to be a conserved mechanism of the immune response (44). These studies suggest that neutral lipid stores could cause a tug of war between a pathogen and its host. The ability of a host to withhold essential micronutrients such as iron and zinc from pathogens, to prevent replication and growth of the latter, is termed nutritional immunity (45). Our study presents a new facet of nutritional immunity or immunometabolism wherein the ability to utilize nutritional reservoirs boosts the host’s immune response and survival.

## MATERIALS AND METHODS

### Strains and growth media.

All bacterial strains used in this study are listed in Table S2 in the supplemental material. E. coli was grown in LB at 37°C for 8 h and seeded on NGM plates with streptomycin. For making pathogen lawns, E. faecalis was grown in BHI with appropriate antibiotics for 5 h at 37°C, and 50 μl was spread on BHI agar plates with gentamycin (50 μg/ml) and kept at 37°C overnight. For some experiments, OG1RF was seeded on NGM agar plates. S. aureus was grown in BHI broth supplemented with 5 μg/ml nalidixic acid followed by plating on a BHI agar plate. P. aeruginosa was grown in LB for 8 to 10 h and spread on SK agar plate (26). *S*. Typhimurium was grown for 4 to 5 h in LB and spread on NGM plate. C. neoformans H99 was grown in yeast extract-peptone-dextrose (YPD) broth for 12 h at 25°C and plated on BHI agar. N2 (Bristol) was used as the C. elegans wild-type strain, and animals were maintained with E. coli OP50 on nematode growth medium (NGM), as previously described (46). All strains used in this study are listed in Table S2. All animals were maintained at 20°C except for temperature-sensitive mutant *daf-2(e1370)*, which was maintained at 15°C. All C. elegans exposures to pathogens were conducted at 25°C unless otherwise mentioned.

### RNA interference.

The C. elegans RNAi library was obtained from Source BioScience. Individual HT115(DE3) bacterial clones expressing double-stranded RNA (dsRNA) against genes of interest were grown at 37°C in LB with carbenicillin (25 μg/ml) and then seeded onto NGM-carbenicillin plates supplemented with 2.5 mM isopropyl-β-d-thiogalactopyranoside (IPTG). Eggs laid on RNAi plates were allowed to develop until the young adult stage. Animals were transferred to a fresh lawn of pathogen prior to lipid staining, RNA extraction, or survival assay.

### C. elegans survival assay.

One hundred to one hundred twenty synchronized young adult animals were exposed to pathogen at 25°C and scored for survival at the times indicated in the survival assay graphs. Animals were considered dead when they stopped moving and failed to respond to touch. Each survival assay was performed three times or more.

### Oil Red O staining and BODIPY staining.

Animals were stained with Oil Red O as described (15). Briefly, a synchronized population of adult C. elegans was fixed by incubating the animals with equal volumes of phosphate-buffered saline (PBS) and 2× MRWB [160 mM KCl, 40 mM NaCl, 14 mM Na_2_EGTA, 1 mM spermidine-HCl, 0.4 mM spermine, 30 mM Na-piperazine-*N*,*N*′-bis(2-ethanesulfonic acid) (PIPES; pH 7.4), 0.2% beta-mercaptoethanol, and 20 mg/ml paraformaldehyde (PFA)] for 1 h at room temperature. Animals were washed three times with PBS and then incubated with 60% isopropanol for 15 min at room temperature. Animals were stained with freshly prepared 60% ORO stain for 16 to 18 h following which they were washed with PBS plus 0.1% Triton X-100. Animals were mounted on 2% agarose pads and imaged by using an Olympus IX81 bright-field microscope. ORO staining in animals was quantified using ImageJ software. ORO stain was quantified in 15 to 20 animals in each experimental set/strain/treatment. All the experiments were performed at least 3 times.

Animals were stained with BODIPY as described previously (47). A synchronized population of adult animals was fixed in 4% paraformaldehyde followed by three cycles of freeze-thaw. Fixed animals were stained in 1 μg/ml BODIPY^493/503^ in M9 buffer for 1 h at room temperature. The stained animals were imaged by using an Olympus IX81 fluorescence microscope. Image analyses and quantification were conducted using ImageJ.

### Carbohydrate and protein estimation.

Carbohydrate content in C. elegans was estimated by the anthrone assay, as described previously (48). Briefly, 800 to 1,000 animals, exposed to OP50 or OG1RF for 8 h, were collected in M9 buffer and sonicated (55% amplitude, pulse on, 1 min; pulse off, 30 s) for 45 min using a Branson digital sonifier. To 100 μl of the sonicated lysate, 500 μl of 0.14% anthrone reagent was added, and the mixture was incubated at 80°C for 20 min. Absorbance was measured at 620 nm using a TECAN Infinite M200pro. Carbohydrate content per animal was calculated based on a glucose standard curve prepared using anthrone reagent. Protein estimation was performed by using a Bradford assay (49). To 20 μl of the sonicated lysate, 40 μl of Bradford reagent and 240 μl of water were added. The reaction mixture was incubated in the dark for 30 min, and absorbance was measured at 595 nm. The protein content of animals was estimated by the Bradford assay against a standard curve of bovine serum albumin. Carbohydrate and protein estimation tests were conducted three independent times.

### Fluorescence microscopy.

Adult *acs-2*P::GFP and adult *fmo-2*P::GFP animals were allowed to feed on OP50 and OG1RF for 8 h or 24 h. Ten to 12 animals were mounted on fresh agarose-pad slides and imaged by using an Olympus IX81 fluorescence microscope. These images were used for quantification using ImageJ. Each experiment was conducted three times or more.

### Quantitative real-time PCR.

A total of 800 to 1,000 synchronized adult animals were exposed to OP50 and OG1RF for 8 h. Animals were collected using M9 buffer and frozen in QIAzol lysis reagent at −80°C. Total RNA was extracted using an RNeasy Plus Universal minikit (Qiagen) and reverse transcribed using an iScript cDNA synthesis kit (Bio-Rad). cDNA was subjected to qRT-PCR analysis using SYBR green detection (iTaq Universal supermix; Bio-Rad) on a QuantStudio3 (Applied Biosystems) machine. Primers for qRT-PCR were designed using Primer 3 online software. All threshold cycle (*C_T_*) values were normalized using a control gene, *act-1* (actin-1). The comparative Δ*C_T_* method was used to determine fold change in gene expression (50). All experiments were repeated at least three times, and fold change data are presented as means ± standard errors of the means (SEMs). Primer sequences are available upon request.

### RNA-sequencing and data analysis.

RNA was isolated from approximately 1,000 L4 animals exposed to OP50 and OG1RF by using a Qiagen Universal RNA isolation kit. Three biological replicates were prepared for each. A cDNA library was prepared using an NEBNext Ultra II Directional RNA Library Prep kit for Illumina. The libraries were subjected to 75-base single-end sequencing using an Illumina sequencer (services from Genotypic Inc.). The data were analyzed using a pipeline described previously (51). HISAT was used to map the RNA-Seq reads onto the latest C. elegans reference genome (WS266) from Wormbase, resulting in a sequence alignment map (SAM). SAM format was converted to its binary form, BAM, using SAMtools. BAM files were then used as input for assembling the transcripts using Stringtie. Stringtie was used to assemble and quantify the levels of expressed genes to produce fragments per kilobase of exon per million fragments mapped (FPKM). The assembly was merged into a singular gene transfer format (GTF) to facilitate comparison with the reference annotation in the same format. Ballgown was used to calculate differential expression of genes using FPKM data and to generate tables with fold change and *P* values. Genes were shortlisted with a cutoff of 2-fold change and *P* values of less than 0.05.

### Gene ontology analysis.

To understand the functional relevance of gene expression changes obtained from RNA-Seq analysis, transcripts upregulated under each condition were categorized using DAVID (Database for Annotation, Visualization and Integrated Discovery), an online software. Wormbase gene identifiers (IDs) of all the genes upregulated more than 2-fold (with *P* value < 0.05) for a particular sample were taken as the input. Gene ontology analysis was performed on the input genes. Gene ontology can cluster the genes under three domains, namely, biological processes (BP), molecular functions (MF), and cellular compartments (CC). We selected BP for our analysis. The input genes clustered under several GO categories as shown in Fig. 1A to C for each sample.

### Statistical analysis.

Survival curves for C. elegans adults were plotted using GraphPad Prizm. The Kaplan-Meier method was used to calculate survival fractions and the log rank test was used to compare survival curves (Table S3). Survival curves were considered different from the appropriate control when *P* values were <0.05. A two-sample *t* test for independent samples was used to analyze Oil Red O, BODIPY, body measurements, fluorescence intensity experiments, and qRT-PCR results. *P* values of <0.05 are considered significant. All experiments were repeated at least three times unless otherwise indicated.

### Data availability.

Raw RNAseq data have been deposited in the NCBI BioProject database under accession numbers PRJNA521879, PRJNA524750, and PRJNA524775.

## Supplementary Material

Supplemental file 1

Supplemental file 2

Supplemental file 3

Supplemental file 4

Supplemental file 5
