# Supplementary figures and images for: NHR-49 Transcription Factor Regulates Immunometabolic Response and Survival of Caenorhabditis elegans during Enterococcus faecalis Infection
Source: Infect Immun. 2020 Jul 21;88(8):e00130-20. doi: 10.1128/IAI.00130-20 (PMC7375755; doi:10.1128/IAI.00130-20)

## GRAPHICAL ABSTRACT

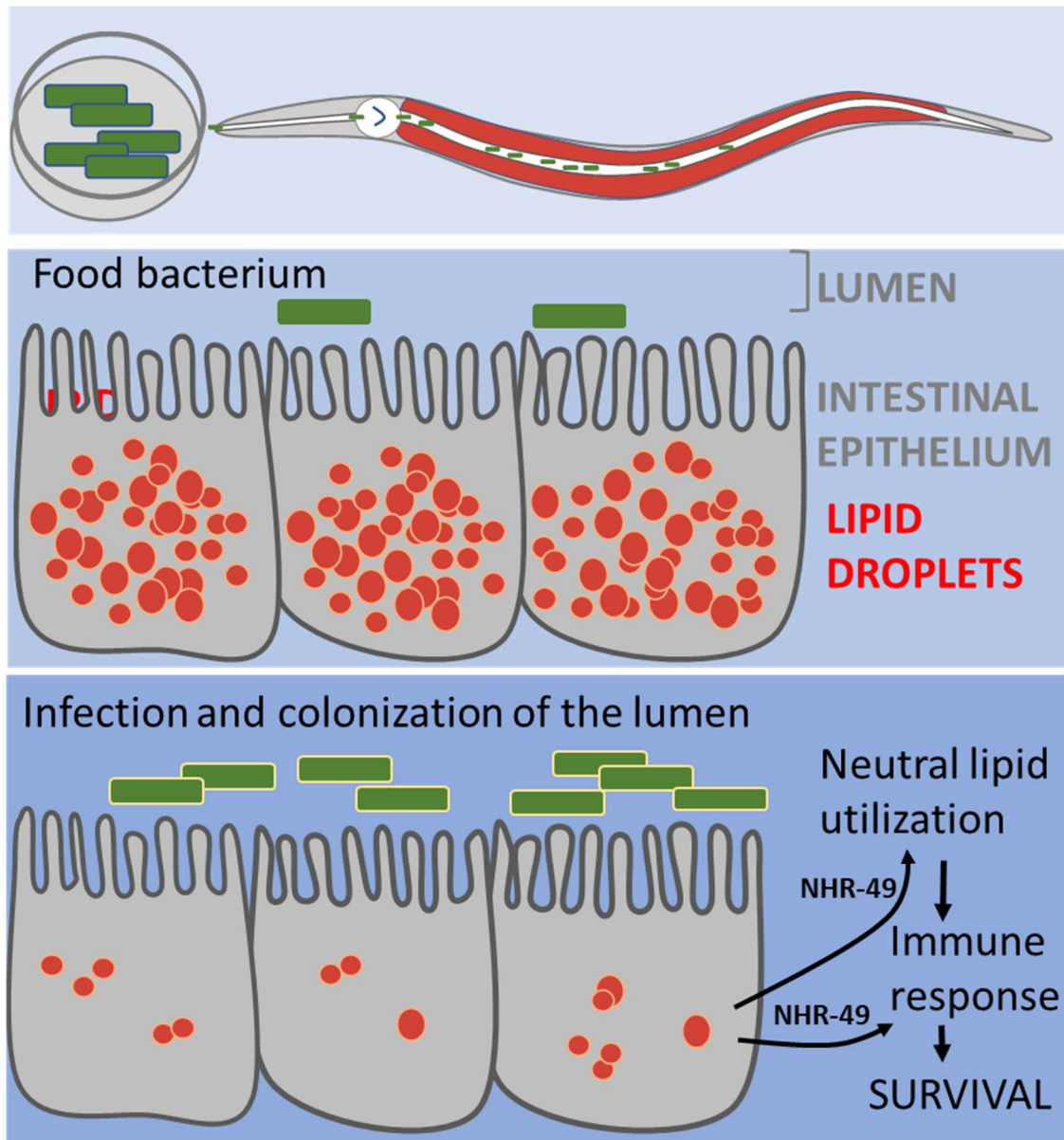

Supplement: Supplemental file 4 [file IAI.00130-20-s0004.pdf]
