# Supplementary material for: NHR-49 Transcription Factor Regulates Immunometabolic Response and Survival of Caenorhabditis elegans during Enterococcus faecalis Infection
Source: Infect Immun. 2020 Jul 21;88(8):e00130-20. doi: 10.1128/IAI.00130-20 (PMC7375755; doi:10.1128/IAI.00130-20)
Supplement: Supplemental file 5 [file IAI.00130-20-s0005.pdf]

FIGURE S1

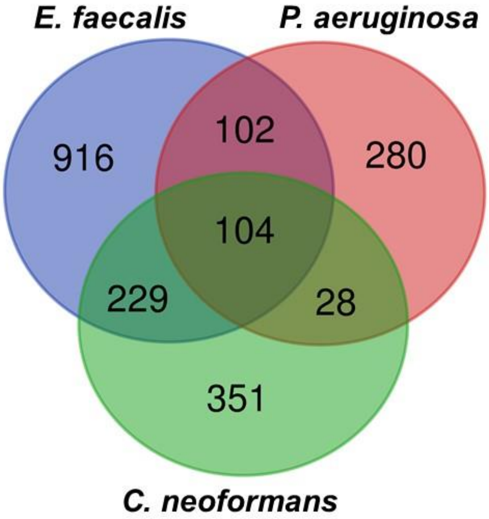

Figure S1 Exposure of *C. elegans* to pathogen for 8 hours induces a transcriptional response. Venn diagram of genes induced more than 2-fold in *C. elegans* fed on *E. faecalis* (OG1RF), *P. aeruginosa* (PA14) and *C. neoformans* (H99).

FIGURE S2

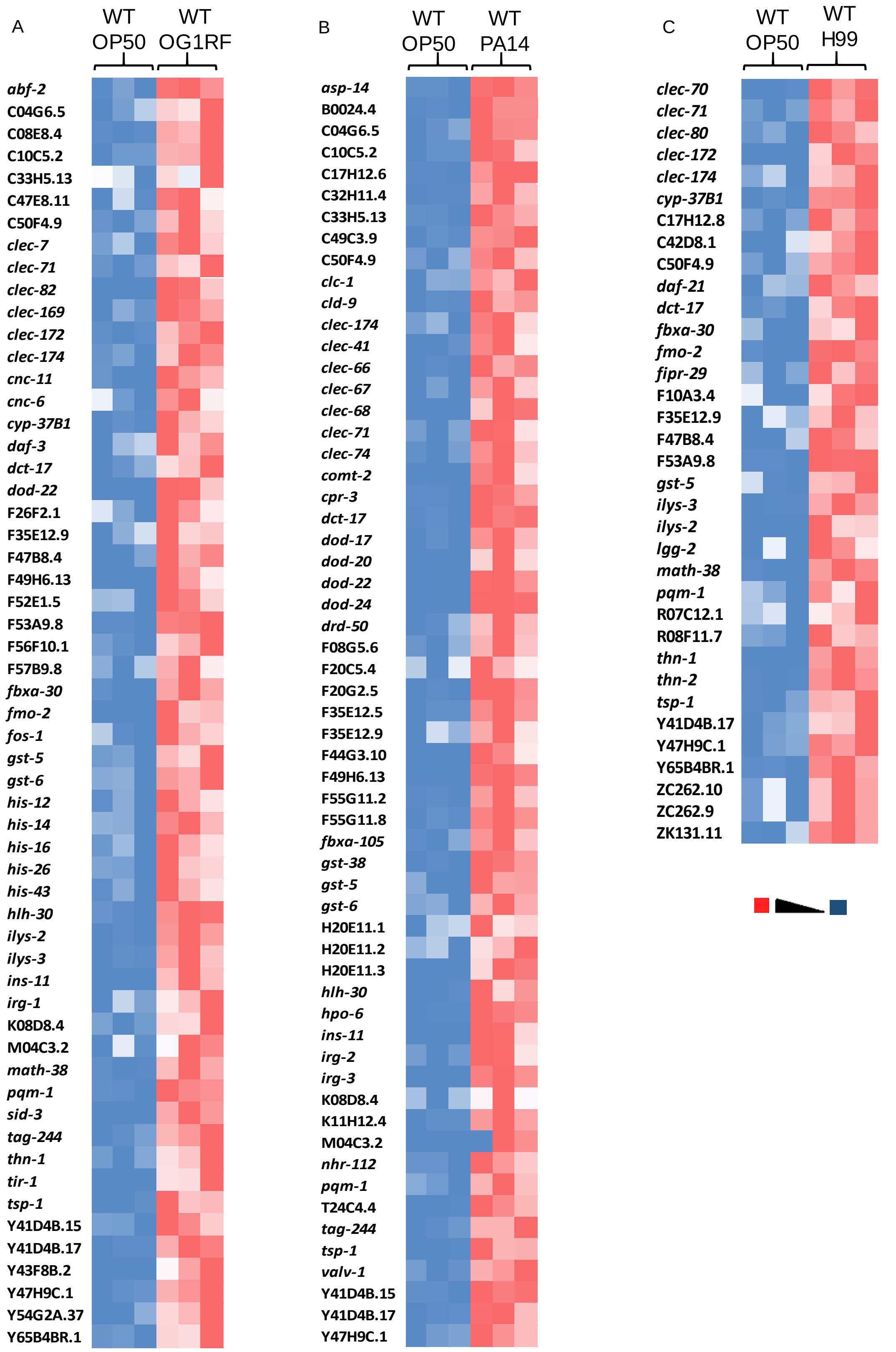

Figure S2 Exposure of *C. elegans* to pathogen diet for 8 hours induces immune effector genes

(A-C) Heat map of expression of immune effector genes in *C. elegans* fed on (A) OG1RF, (B) PA14 and (C) H99 for 8 hours with respect to animals fed on OP50.

FIGURE S3

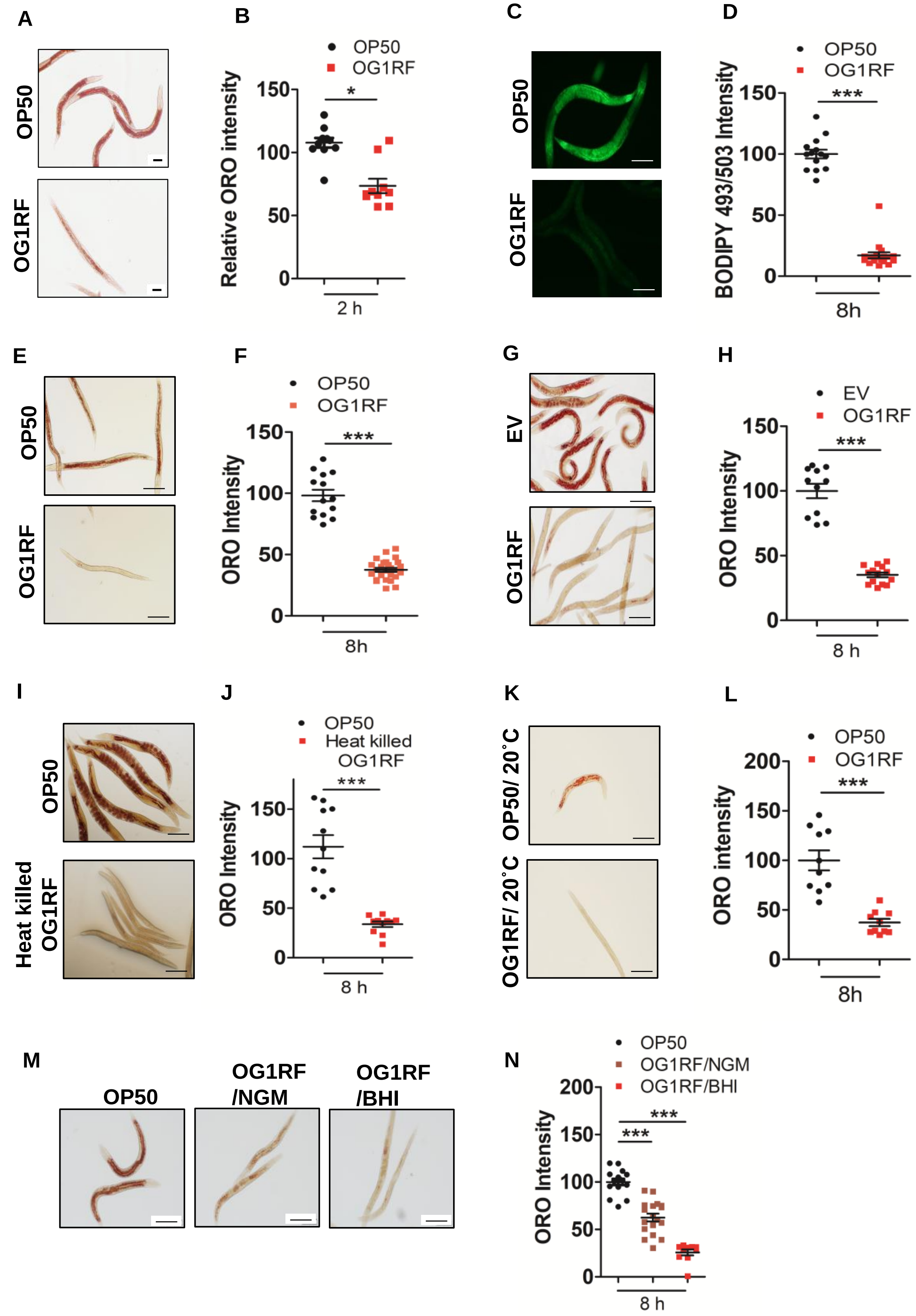

A
OP50
OG1RF
B
OP50
OG1RF
Relative ORO intensity
150
100
50
0
*
2 h
C
OP50
OG1RF
D
OP50
OG1RF
BODIPY 493/503 Intensity
***
8h
E
OP50
OG1RF
F
OP50
OG1RF
ORO Intensity
***
8h
G
EV
OG1RF
H
EV
OG1RF
ORO Intensity
***
8 h
I
OP50
Heat killed OG1RF
J
OP50
Heat killed OG1RF
ORO Intensity
***
8 h
K
OP50/ 20˚C
OG1RF/ 20˚C
L
OP50
OG1RF
ORO intensity
200
***
8h
M
OP50
OG1RF /NGM
OG1RF /BHI
N
OP50
OG1RF/NGM
OG1RF/BHI
ORO Intensity
***
***
8 h

Figure S3 Gram positive cocci *E. faecalis* induces lipid droplet depletion in *C. elegans* upon 8 hour exposure

(A-B) ORO staining of lipids and quantification in adult *C. elegans* fed on OG1RF for 2 hours. (C-D) BODIPY staining of lipids and quantification in adult *C. elegans* fed on OG1RF. (E-F) ORO staining of lipids and quantification in 40-hour old animals fed on OG1RF. (G-H) ORO staining and quantification in germline less (*cdc-25.1* RNAi) animals fed on OG1RF. (I-J) ORO staining and quantification in animals fed on heat killed OG1RF. (K-L) ORO staining and quantification in animals fed on OG1RF at 20˚C. (M-N) ORO staining and quantification in animals fed on OG1RF diet grown on NGM and BHI.

Scale bar, 100 μm. Data is represented as Mean ± SEM. n = 15-20 animals/ condition/ experiment. *** indicates p<0.0001) (n = 15-20 worms/ sample).

FIGURE S4

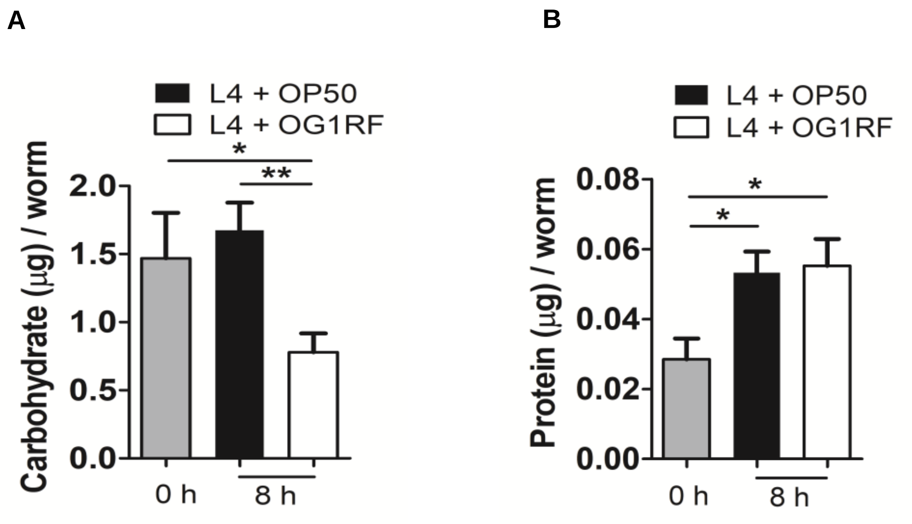

Figure S4 *E. faecalis* diet results in reduction in carbohydrate content in *C. elegans.* (A) Carbohydrate and (B) protein estimation for L4 animals fed OP50 and OG1RF for 8 hours. Data is represented as Mean ± SEM. n = 1000 worms/ sample/ experiment

FIGURE S5

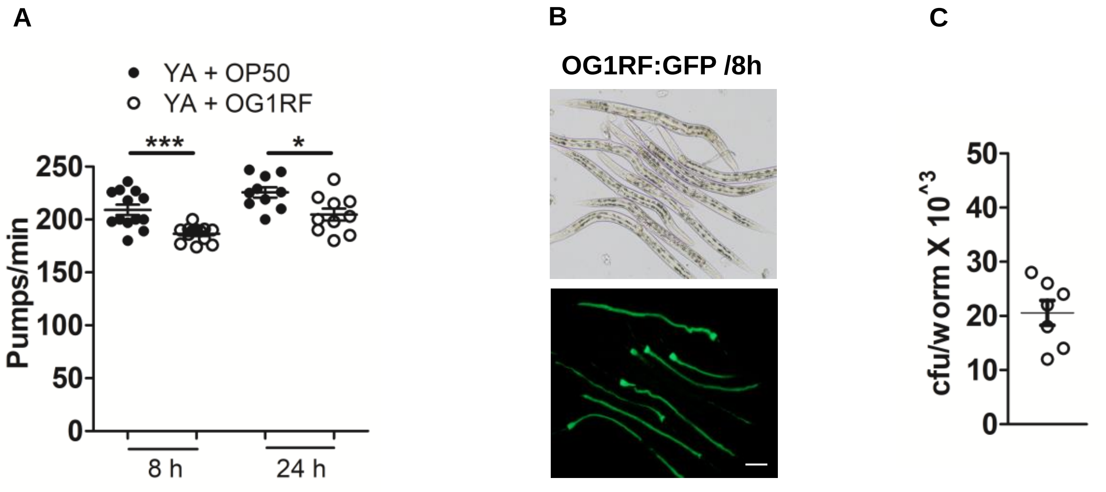

Figure S5 *C. elegans* feeds on *E. faecalis* diet

(A) Number of pharyngeal pumps per minute were measured for young adult animals (YA, 56 hour old animals) feeding OP50 or OG1RF for 8 and 24 hours. Data is represented as Mean ± SEM. n = 10 animals/ sample/ experiment. (B) Images of N2 fed on OG1RF-GFP for 8 hours. (C) Number of bacterial colonies recovered from each animal after 8 hours of feeding on OG1RF-GFP. Data is represented as Mean ± SEM n = 10 animals/ sample/ experiment. Scale bar represents 100μm.

Significance has been measured using unpaired t test. *** indicates $p < 0.0001$, ** indicates $p < 0.05$

FIGURE S6

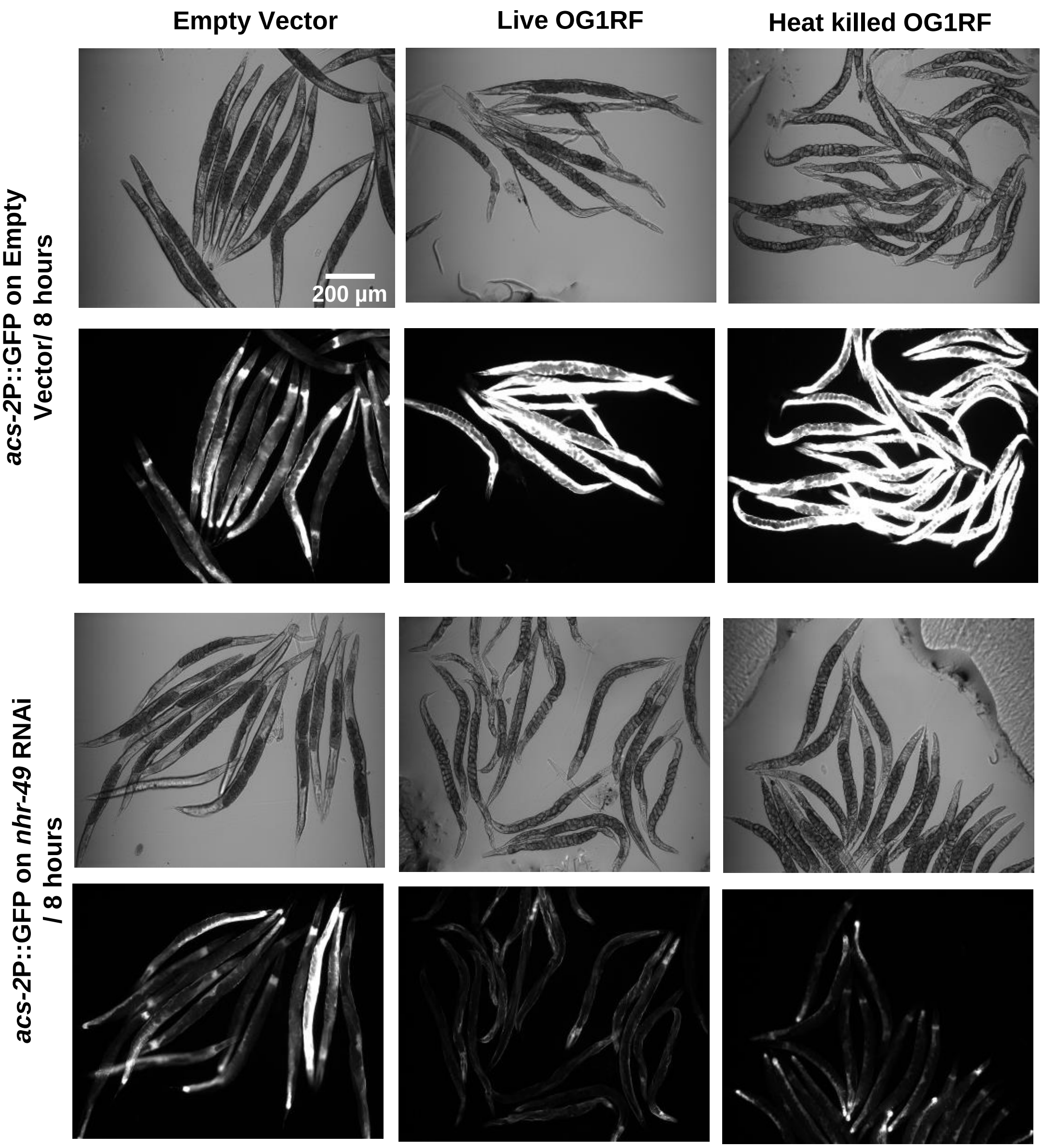

Figure S6 Heat-killed *E. faecalis* diet induces *nhr-49*-regulated *acs-2*P::GFP expression.
L4 *acs-2*P::GFP animals grown on empty vector (top panel) and *nhr-49* RNAi (bottom panel), exposed to live or heat-killed *E. faecalis* (OG1RF) for 8 hours.

FIGURE S7

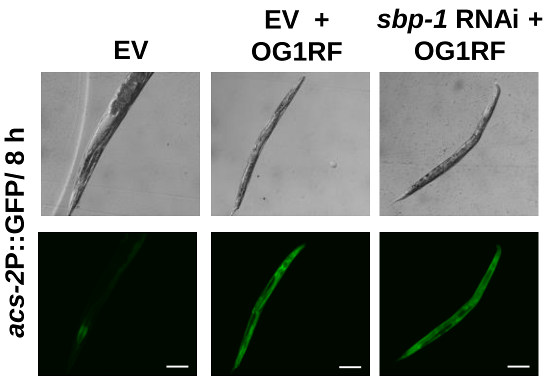

Figure S7 SBP-1 does not regulate expression levels of ACS-2 induced in *C. elegans* fed on *E. faecalis*

Effect of RNAi of *sbp-1* on *acs-2P::GFP* fluorescence upon feeding on OG1RF for 8 hours. Scale bar, 100 μm.

FIGURE S8

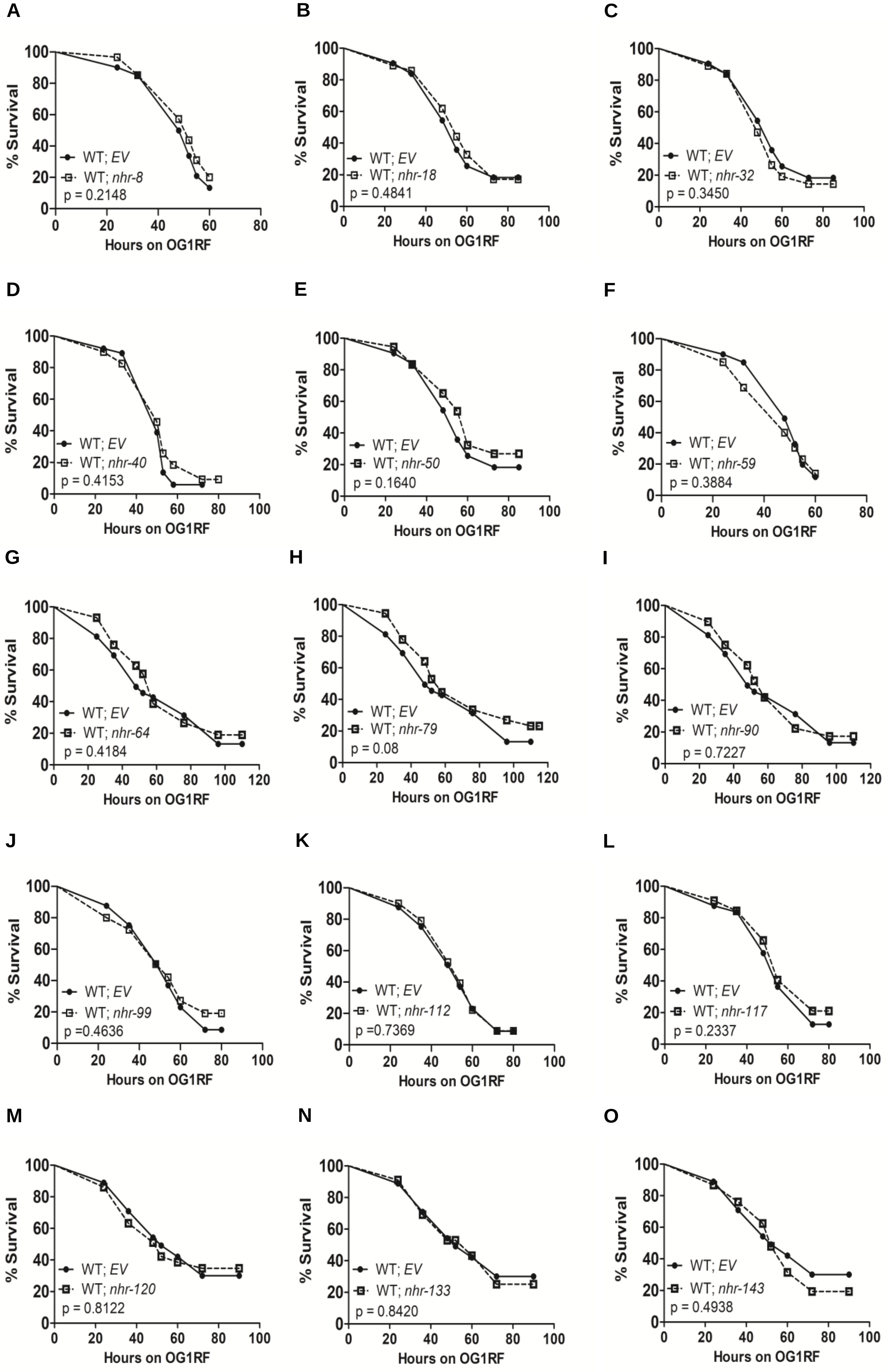

A
% Survival
Hours on OG1RF
WT; *EV*
WT; *nhr-8*
p = 0.2148
B
% Survival
Hours on OG1RF
WT; *EV*
WT; *nhr-18*
p = 0.4841
C
% Survival
Hours on OG1RF
WT; *EV*
WT; *nhr-32*
p = 0.3450
D
% Survival
Hours on OG1RF
WT; *EV*
WT; *nhr-40*
p = 0.4153
E
% Survival
Hours on OG1RF
WT; *EV*
WT; *nhr-50*
p = 0.1640
F
% Survival
Hours on OG1RF
WT; *EV*
WT; *nhr-59*
p = 0.3884
G
% Survival
Hours on OG1RF
WT; *EV*
WT; *nhr-64*
p = 0.4184
H
% Survival
Hours on OG1RF
WT; *EV*
WT; *nhr-79*
p = 0.08
I
% Survival
Hours on OG1RF
WT; *EV*
WT; *nhr-90*
p = 0.7227
J
% Survival
Hours on OG1RF
WT; *EV*
WT; *nhr-99*
p =0.4636
K
% Survival
Hours on OG1RF
WT; *EV*
WT; *nhr-112*
p =0.7369
L
% Survival
Hours on OG1RF
WT; *EV*
WT; *nhr-117*
p = 0.2337
M
% Survival
Hours on OG1RF
WT; *EV*
WT; *nhr-120*
p = 0.8122
N
% Survival
Hours on OG1RF
WT; *EV*
WT; *nhr-133*
p = 0.8420
O
% Survival
Hours on OG1RF
WT; *EV*
WT; *nhr-143*
p = 0.4938

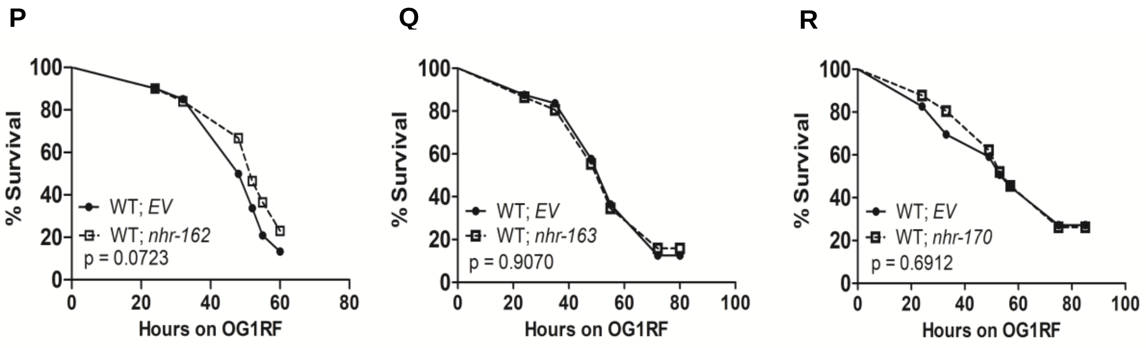

Figure S8 Screening nuclear hormone receptors for their role in promoting survival in *C. elegans* during *E. faecalis* infection

Kaplan Meier survival curve of (A) *nhr-8* RNAi (B) *nhr-18* RNAi (C) *nhr-32* RNAi (D) *nhr-40* RNAi (E) *nhr-50* RNAi (F) *nhr-59* RNAi (G) *nhr-64* RNAi (H) *nhr-79* RNAi (I) *nhr-90* RNAi (J) *nhr-99* RNAi (K) *nhr-112* RNAi (L) *nhr-117* RNAi (M) *nhr-120* RNAi (N) *nhr-133* RNAi (0) *nhr-143* RNAi (P) *nhr-162* RNAi (Q) *nhr-163* RNAi and (R) *nhr-170* RNAi in wild type animals fed on OG1RF. n = 100 animals/ strain/ experiment

FIGURE S9

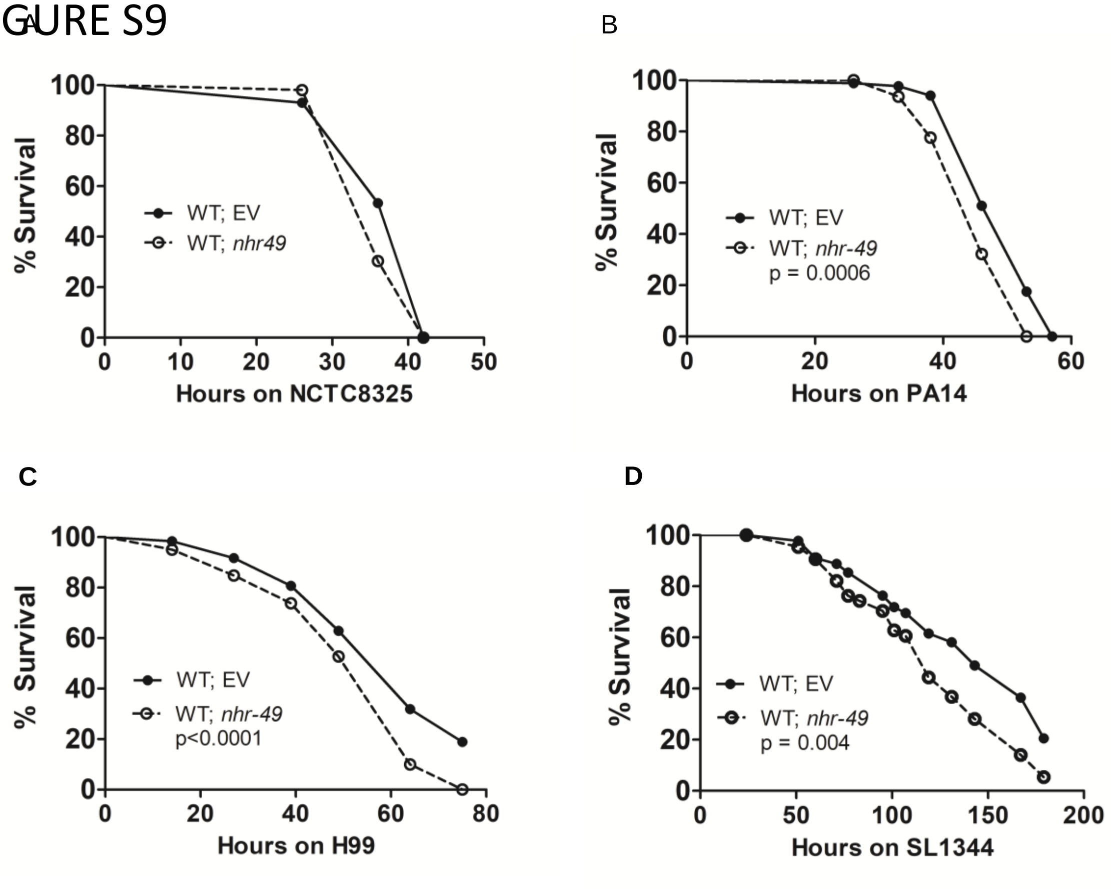

Figure S9 NHR-49 positively regulates survival of wild type animals upon infection

(A-D) Kaplan Meier survival curves of *nhr-49* RNAi animals and control animals exposed to (A) *S. aureus* NCTC8325, (B) *P. aeruginosa* PA14 (p=0.0006), (C) *C. neoformans* (p<0.0001). (D) *S. typhimurium* (p=0.004).

FIGURE S10

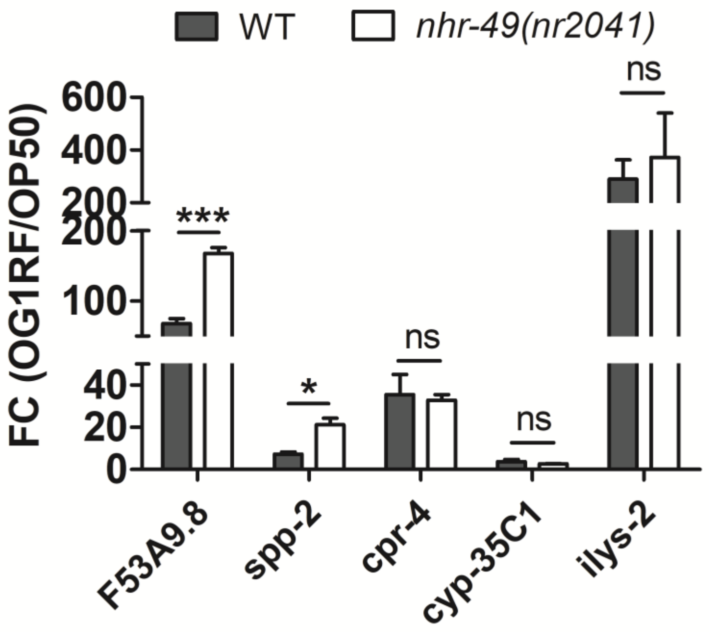

Figure S10 Expression levels of immune effectors in *nhr-49(nr20141)* mutant fed on *E. faecalis* OG1RF induced transcript levels of *F53A9.8* and *spp-2* (encoding for Saposin like protein) were significantly downregulated in *nhr-49(nr2041)* mutant compared to wild type. Data is represented as Mean ± SEM. N= 3 independent experiments. *** indicates p <0.001

FIGURE S11

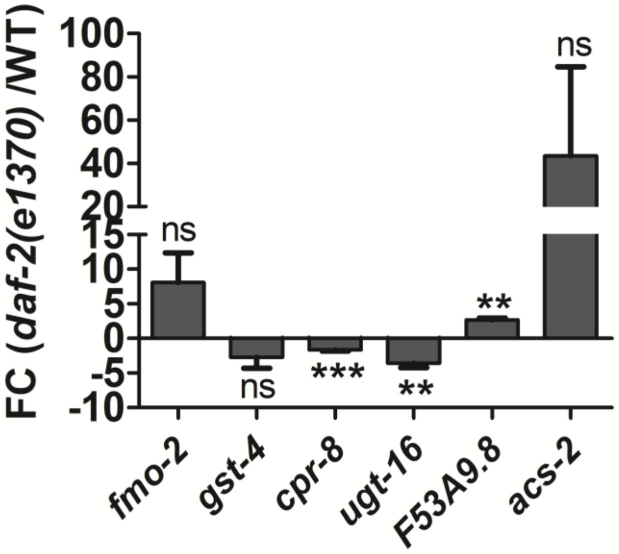

Figure S11 Basal levels of immune effectors in *daf-2(e1370)* mutant Transcript levels of *fmo-2* was significantly dysregulated in *daf-2* mutant worms. Data is represented as Mean ± SEM. N= 3 independent experiments. * indicates p <0.01
